# Decoding Periorbital Aging: A Multilayered Analysis of Anatomical Changes

**DOI:** 10.1007/s00266-024-04590-1

**Published:** 2025-01-08

**Authors:** Pongsak Lohakitsatian, Padcha Tunlayadechanont, Thiti Tantitham

**Affiliations:** 1 Division of Plastic and Maxillofacial Surgery, Department of Surgery, Ramathibodi Hospital, Mahidol University, Bangkok, Thailand; 2 Division of Neuroimaging, Department of Diagnostic and Therapeutic Radiology, Ramathibodi Hospital, Mahidol University, Bangkok, Thailand

**Keywords:** Orbital aging, Orbital fat, Periorbital rejuvenation

## Abstract

**Background:**

Periorbital aging is a complex phenomenon that involves multiple layers of facial anatomy, including bone, fat, and globe. While previous studies have predominantly focused on age-related changes in facial fat compartments, this research aims to provide a comprehensive understanding of all periorbital components, including upper and lower orbital fat, orbital cavity volume, globe volume, and globe position, in the context of aging.

**Methods:**

We conducted a retrospective study involving 118 patients (236 subjects) aged 18–99 years who underwent brain MRI using a 3 Tesla MR system. Baseline demographics and various parameters pertaining to periorbital aging were collected, and comprehensive measurements were obtained through meticulous radiological analysis.

**Results:**

Our findings revealed distinct patterns of age-related changes in the periorbital region. Upper orbital fat remained stable with age, while lower orbital fat exhibited a substantial increase in both anterior and posterior compartments. Notably, orbital cavity volume expanded with bony resorption, while eye globe volume decreased, contributing to an enophthalmic appearance. We observed no vertical displacement of the globe with aging.

**Conclusion:**

This study provides a comprehensive overview of the multifaceted anatomical changes that occur in the periorbital region with aging. The insights gained from this research offer important clinical implications for addressing the signs of periorbital aging, guiding surgical interventions, and ultimately enhancing patient outcomes.

**Level of Evidence IV:**

This journal requires that authors assign a level of evidence to each article. For a full description of these Evidence-Based Medicine ratings, please refer to the Table of Contents or the online Instructions to Authors www.springer.com/00266.

## Introduction

Periorbital aging is a complex process that involves all facial layers, ranging from the deep layer of bone to the superficial layer of skin. Age-related changes in bone structure, particularly the resorption of the superomedial and inferolateral rim, have been extensively documented [1, 2]. These changes result in distinct signs of aging, such as superior sulcus hollowness and tear trough deformity. While numerous studies have focused on the redistribution of facial fat compartments [3–5], the focus in most investigations concerning orbital fat change has been limited to inferior orbital fat [6–9]. Notably, orbital volume is observed to increase with age, which causes the orbital contents to retract deeper into the orbital vault, leading to a more enophthalmic appearance, which appears to primarily impact the upper eyelid hollowness and not the protrusion of lower eyelid fat [10–12]. We initially hypothesized a more complex interaction between the various periorbital structures. The age-related increase in orbital volume may contribute to globe descent, which pushes the lower orbital fat forward while causing retrusion of the upper orbital fat. Based on this concept, upper blepharoplasty primarily focuses on preserving volume, whereas lower blepharoplasty emphasizes volume reduction or redistribution. We contend that achieving effective periorbital rejuvenation surgery necessitates a comprehensive understanding of the changes in all periorbital components that occur with aging. To gain insights into periorbital aging, a detailed examination of each component of periorbital anatomy, including the bony orbit, the globe, and fat, is necessary, along with the interplay between these structures. This study aims to investigate changes in orbital fat volume, orbital cavity volume, globe volume, and globe position in relation to aging. Only by understanding each piece of the puzzle can we assemble the larger picture of periorbital aging.

## Materials and Methods

**Fig. 1.**
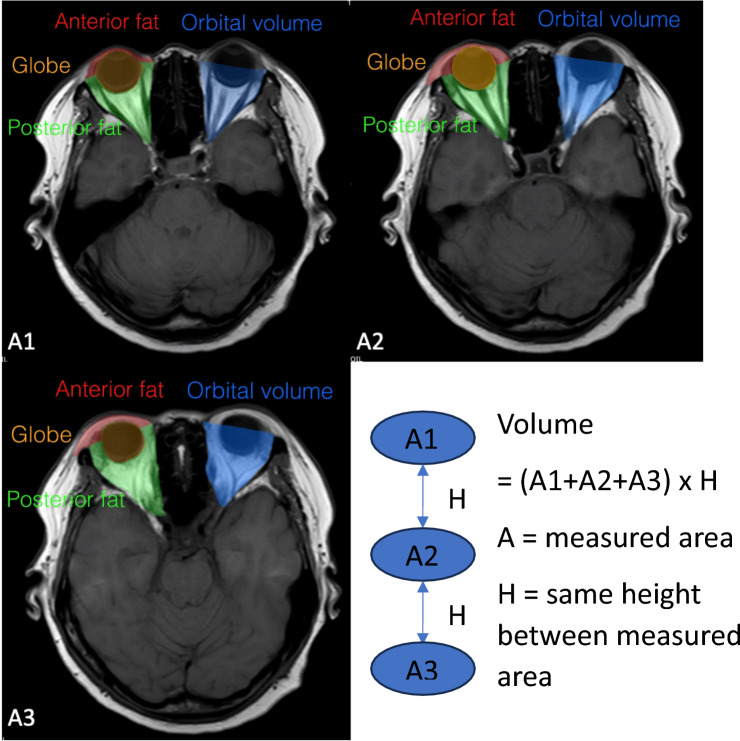
Measurement from axial view of MRI and formula for volume calculation

**Fig. 2.**
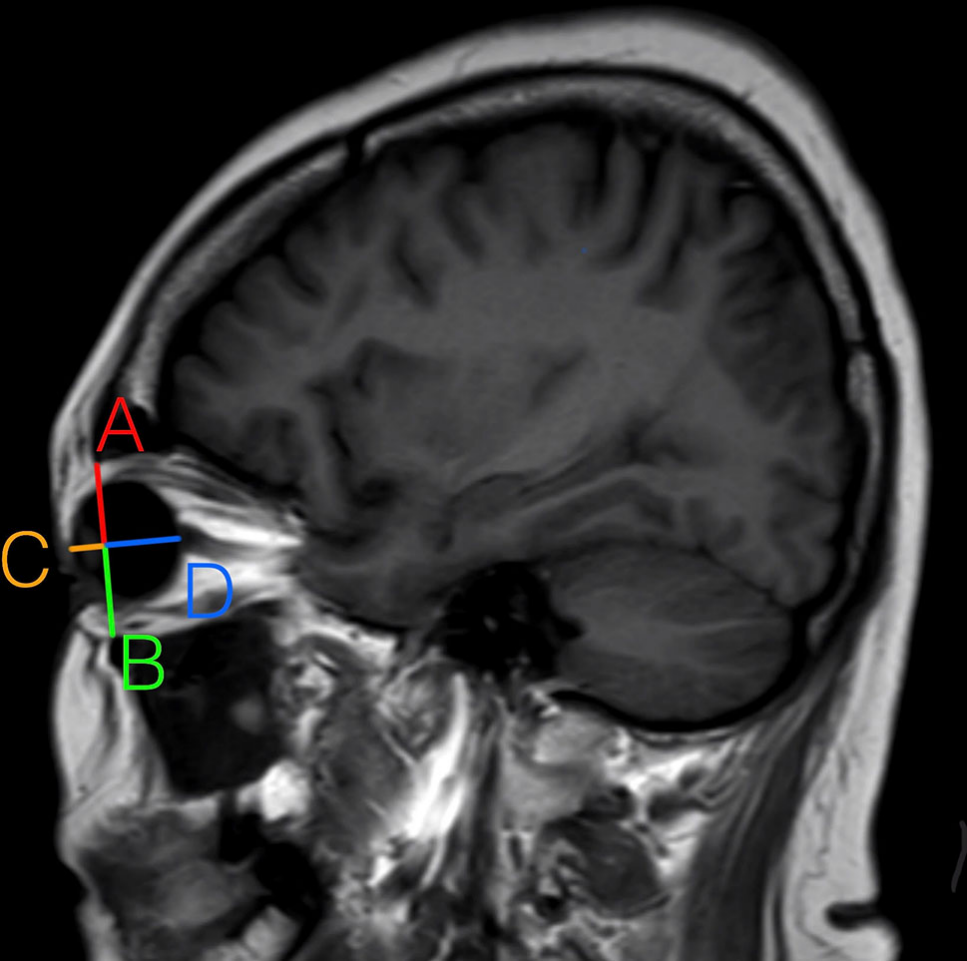
Sagittal view of MRI and globe position in anteroposterior and vertical positions

A retrospective study was conducted by reviewing patients aged 18–91 years who underwent brain MRI (magnetic resonance imaging) using a 3-Tesla MR system (Philips Ingenia; Philips Medical Systems, Best, Netherlands) with a standard head coil. The routine brain MRI protocol included T1-weighted and T2-weighted sequences (FOV 240x240 mm; matrix 240x240 mm; slice thickness/gap 4/4 mm). Data were collected from January 2019 to May 2020, with exclusion criteria including primary ophthalmologic diseases, periorbital infection, craniofacial tumors, craniofacial anomalies, chronic kidney disease stage ≥ 3, a history of thyroid disease, prior periorbital surgery, periorbital trauma, periorbital radiation therapy, and a BMI exceeding 30. An institutional review board (IRB) approved this study. Baseline demographic information, including age, gender, and BMI, was collected. Parameters related to periorbital aging encompassed upper anterior orbital fat volume, upper posterior orbital fat volume, lower anterior orbital fat volume, lower posterior orbital fat volume, total upper orbital fat volume, total lower orbital fat volume, total orbital fat volume, orbital cavity volume, globe volume, and globe position. Prior to data collection, the author received training from a neuroimaging radiologist, followed by agreement measurements and correlation analysis, reflecting excellent intra-rater reliability with a test score of 0.95. Each patient's eye was analyzed separately. A total of 118 patients (236 subjects) were analyzed to investigate the relationship between age, sex, BMI, and the selected parameters. The orbital fat volume referred to in the study pertains to both extraconal fat and intraconal fat, which was segmented into upper anterior orbital fat volume, upper posterior orbital fat volume, lower anterior orbital fat volume, lower posterior orbital fat volume, total upper orbital fat volume, total lower orbital fat volume, and total orbital fat volume. Measurements were taken from the axial view of MRI and then multiplied by height (Fig. 1). Orbital cavity volume and globe volume were measured using a similar methodology. Globe position was assessed separately in anteroposterior (AP) and vertical positions by measuring the distance from the sagittal view of MRI, followed by the calculation of the position ratio (Fig. 2).

Statistical analysis was carried out using STATA version 14 (StataCorp, College Station, Texas, USA). Normally distributed continuous variables were presented as mean ± standard deviation and compared using ANOVA. Categorical data were expressed as frequencies and percentages. A *P *value of < 0.05 was considered statistically significant. Factors associated with the volume of orbital fat, orbital cavity volume, globe volume, and globe position were identified through a nonlinear regression model based on the relationship between volume and age, sex, and BMI. Estimations of these parameters and the corresponding 95 percent confidence intervals (95% CI) were obtained using nonlinear least squares, with estimated s.e. adjusted for clustering around individuals. A *P *value of < 0.05 was considered statistically significant, and only significant predictors were included in the final model. A mixed-effects ML regression was employed for linear regression analysis to examine the factors associated with the volume of orbital fat, orbital cavity volume, globe volume, and globe position.

## Results

A total of 236 eyes from 118 patients were included in the study. The patients' ages ranged from 18 to 91 years, with a mean age of 48.36 ± 20.51 years. Of these patients, 79 were female (66.95%) and 39 were male (33.05%). The average BMI was 22.99 ± 3.42. Univariate linear regression was utilized to investigate the relationship between age and the volume of orbital fat, orbital cavity volume, globe volume, and globe position.

### Upper Orbital Fat Volume

**Fig. 3:**
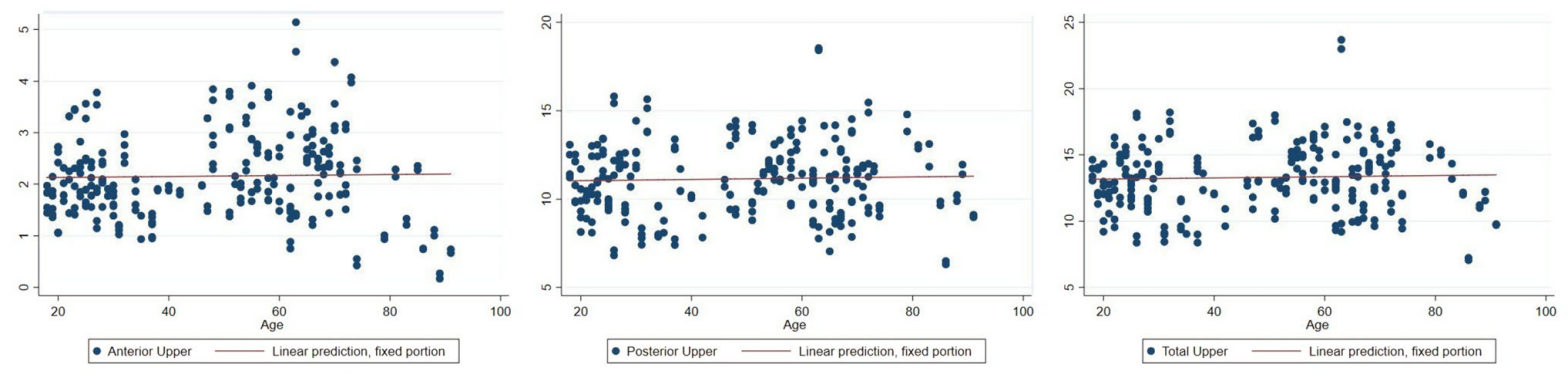
Relationship between upper orbital fat volume and age

There was no statistically significant correlation between age and the volume of anterior, posterior, or total upper orbital fat (Table 1 and Fig. 3).

**Table 1:** Relationship between upper orbital fat volume and age

Variables	Coef.	95% CI	Std. Err.	*P* value
Anterior upper fat	0.0010	− 0.0042, 0.0061	0.0026	0.717
Posterior upper fat	0.0036	− 0.0093, 0.0165	0.0066	0.583
Total upper fat	0.0046	− 0.01111, 0.0203	0.0080	0.568

### Lower Orbital Fat Volume

**Fig. 4:**
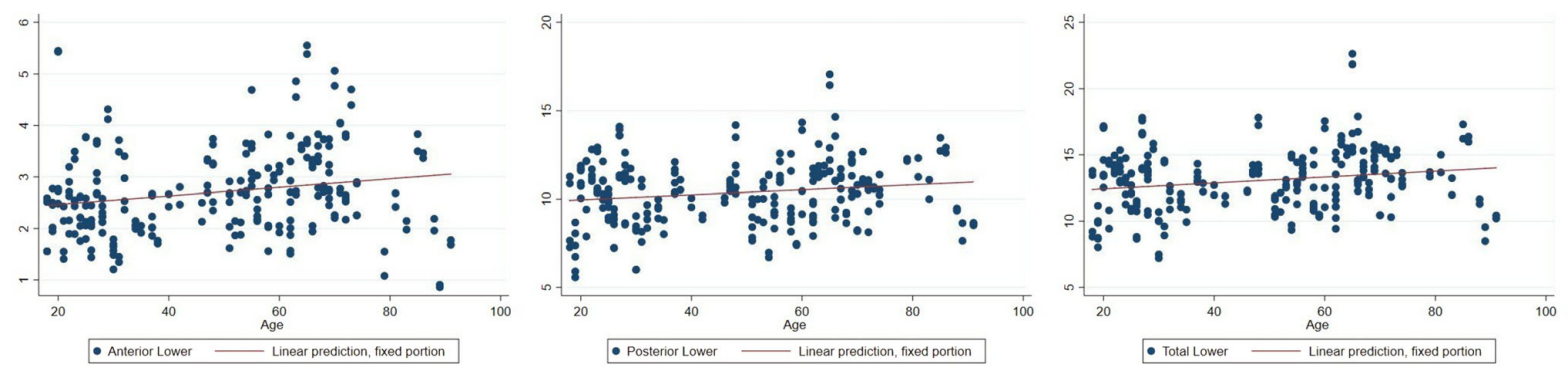
Relationship between lower orbital fat volume and age

An increase in fat volume in the anterior, posterior, and total lower orbital fat volume was observed in correlation with advancing age (Table 2 and Fig. 4).

**Table 2:** Relationship between lower orbital fat volume and age

Variables	Coef.	95% CI	Std. Err.	*P* value
Anterior lower fat	0.0083	− 0.0031, 0.0135	0.0027	**0.002**
Posterior lower fat	0.0141	− 0.0029, 0.0253	0.0057	**0.014**
Total lower fat	0.0224	− 0.0082, 0.0365	0.0072	**0.002**

### Total Orbital Fat Volume

**Fig. 5:**
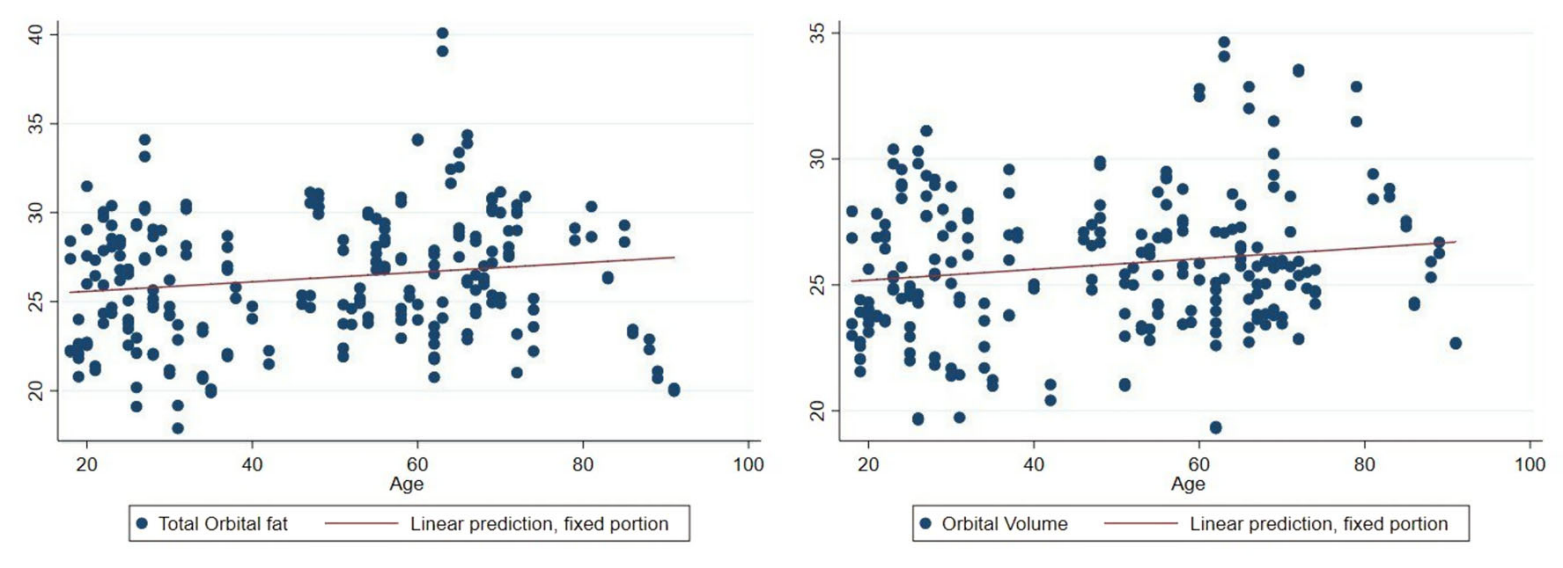
Relationship between total orbital fat volume and age. (Left) Relationship between orbital cavity volume and age. (Right)

Total orbital fat volume was found to increase in relation to aging with statistical significance (Table 3 and Fig. 5).

**Table 3:** Relationship between total orbital fat volume and orbital cavity volume with age.

Variables	Coef.	95% CI	Std. Err.	*P* value
Total orbital fat	0.0269	− 0.0047, 0.0491	0.0113	**0.017**
Orbital cavity volume	0.0214	0.0037, 0.0390	0.0090	**0.018**

### Orbital Cavity Volume

Orbital cavity volume was observed to increase in relation to aging with statistical significance. (Table 3 and Fig. 5).

### Globe Volume

**Fig. 6:**
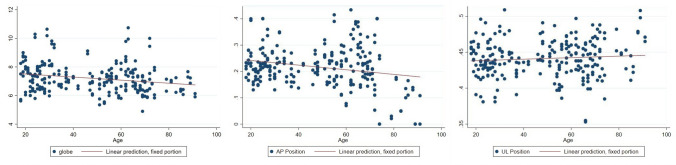
Relationship between eye globe volume and age. (Left) Relationship between globe position in AP dimension and age. (Center) Relationship between globe position in vertical dimension and age. (Right)

Eye globe volume exhibited a significant decrease with age (Table 4 and Fig. 6).

**Table 4:** Relationship between eye globe volume, globe position in AP and vertical dimension with age

Variables	Coef.	95% CI	Std. Err.	*P* value
Globe volume	− 0.0108	− 0.0170, − 0.0045	0.0032	**0.001**
AP position	− 0.0001	− 0.0014, 0.0004	0.0002	**0.000**
Vertical position	0.0001	− 0.0001, 0.0003	0.0001	0.197

### Globe Position

There was a decrease in the anteroposterior (AP) dimension of globe position with age, indicating an enophthalmic tendency. However, there was no change in globe position in the vertical plane (Table 4 and Fig. 6).

**Fig. 7:**
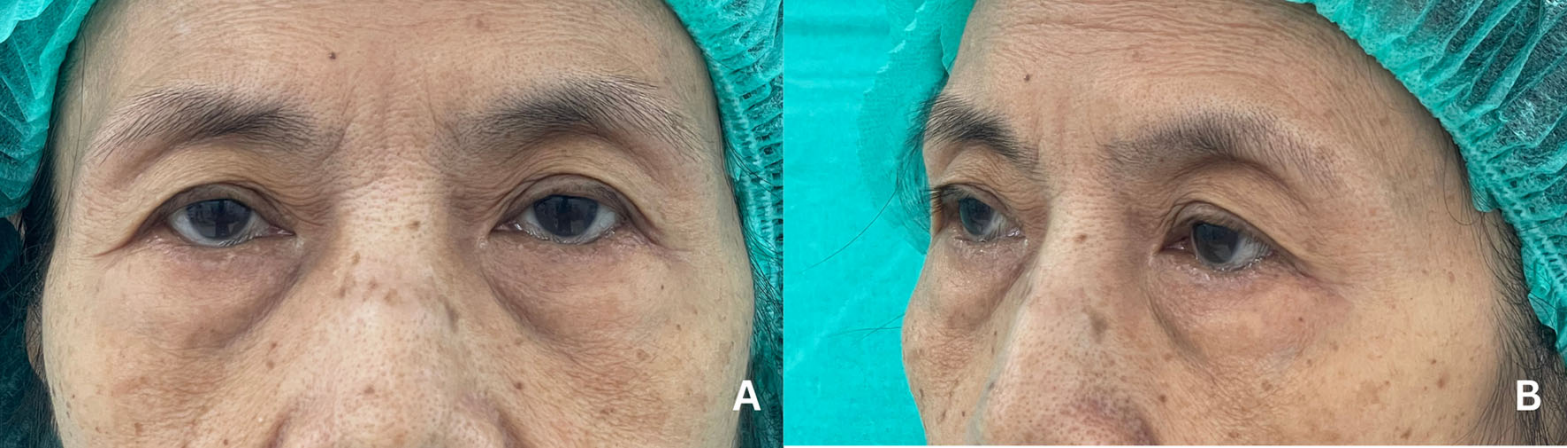
A photograph of a 69-year-old female patient illustrates all the periorbital aging changes identified in our findings, including superior sulcus hollowness, baggy eyelids, and an enophthalmic appearance. **a** Frontal view. **b** Oblique view

Mixed-effects ML regression was used for multivariate analysis involving the side of eyes, age, sex, and BMI in association with the volume of orbital fat, orbital cavity volume, globe volume, and globe position. The results revealed that an increase in BMI was associated with an increase in orbital fat volume, as well as orbital cavity volume, and an increase in the AP dimension of globe position or exophthalmic tendency. No significant difference was found between the two sides of the eyes. Finally, males exhibited larger volumes of posterior upper, total upper, posterior lower, total lower, and total orbital fat than females (Table 5).

## Discussion

Our study has unveiled a comprehensive array of changes in all anatomical structures within the orbit that are associated with aging. Notably, this is the first study to elucidate the alterations and interplay occurring in both upper and lower orbital fat. It is worth emphasizing that not all fat is created equal. While upper orbital fat appears to stabilize as we age, the lower orbital fat increases in volume at both the anterior and posterior compartments to the orbital rim. A similar phenomenon of fat redistribution is observed in midfacial fat, as previously demonstrated by Gierloff et al. [3]. This redistribution involves an inferior volume shift of nasolabial fat, medial cheek fat, and deep medial cheek fat in relation to aging, similar to inferior volume shift found in orbital fat. Our study also uncovered that orbital cavity volume increases with age, in contrast to the orbital globe, which decreases with age. This may contribute to the enophthalmic feature observed in periorbital aging. Interestingly, the bony resorption at the superomedial and inferolateral rims of the orbit does not result in any change in the vertical position of the eye globe, contrary to our initial suspicion.

These findings offer valuable insights into the etiology of various periorbital aging features. A photograph of a 69-year-old female patient illustrates all the periorbital aging changes identified in our findings, including superior sulcus hollowness, baggy eyelids, and an enophthalmic appearance (Fig. 7). The superior sulcus hollowness observed in the upper eyelids can be attributed to the increased orbital cavity volume, despite the stabilized volume of upper orbital fat. This hollowness is further exacerbated by the enophthalmic tendency that accompanies aging. To counteract this aging process, volume augmentation is necessary around the upper eyelids, along with conservative management of upper orbital fat during upper blepharoplasty. The "baggy eyelids" in the lower region are the result of a combination of factors. While Kim J. et al. have identified bony resorption as the primary cause of baggy eyelids [13], we believe that both bony resorption and an increase in lower orbital fat volume are significant contributors. Despite the increased orbital cavity volume, the lower orbital fat appears to hypertrophy at a higher rate. Thus, in lower blepharoplasty, resection or transposition of lower orbital fat is warranted.

Our finding of an increase in bony orbital cavity volume with aging aligns with the results of several previous studies [10–12]. Our study also reveals that eye globe volume decreases with age. Regarding globe position, while Darcy et al. [7] did not find any significant displacement with aging, our study observed a reduction in AP dimension, resulting in slight enophthalmos and no vertical displacement. Our findings on age-related changes in lower orbital fat are consistent with the results of Lee et al. [8]. Not only does lower orbital fat increase with age, but it also protrudes beyond the inferior orbital rim, contributing to the appearance of baggy eyelids. Regensburg, N.I., et al. reported an increase in total orbital fat volume with advancing age, which aligns with our findings. However, while they observed no change in orbital volume, our study found that orbital volume increases with aging. Interestingly, Regensburg, N.I., et al. found no change in orbital muscle volume with advancing age [11] . Additionally, our results offer new objective insights into the upper orbital fat, globe position, and globe volume.

BMI and gender emerged as independent factors affecting several parameters in our analysis. An increase in BMI was associated with increased orbital fat volume across all compartments, subsequently leading to an enlarged orbital cavity volume and an appearance of exophthalmos. Moreover, males exhibited larger orbital fat volumes than females [11].

Our study aims to offer a comprehensive understanding of periorbital changes related to aging. However, there are several limitations. Racial differences may play a role in age-related changes. For instance, Von Lanz et al. [14] found that Caucasian adults have a larger orbital volume compared to Japanese adults, suggesting that Asians tend to have shallower orbits. The volume measurement of posterior orbital fat includes not only fat but also muscles and nerves, making it nearly impossible to accurately distinguish these structures on MRI. Obtaining MRI scans from the same patient across each decade of life would greatly enhance our ability to thoroughly understand these age-related changes.

## Conclusion

The aging process impacts every anatomical structure in the periorbital region. Notably, orbital fat does not exhibit uniform changes with age. Specifically, there is an increase in the volume of lower orbital fat, whereas the volume of upper orbital fat remains stable. The increase in orbital cavity volume, attributed to bony resorption, coincides with a decrease in eye globe volume and a reduction in the anteroposterior (AP) dimension of the orbit, contributing to the enophthalmic appearance associated with aging. Understanding the intricate interplay between these anatomical structures offers valuable guidance to surgeons performing periorbital rejuvenation procedures.

## Appendix A: Factors Associated with the Parameters

See Table 5.

**Table 5:** Mixed-effects ML regression for multivariate analysis between side of eyes, age, sex, and BMI in association with the volume of orbital fat, volume of orbital cavity, volume of globe, and position of globe

Variable		Univariate			Multivariate		
Outcome Y	X	Coef.	95% CI	*P *value	Coef.	95% CI	*P *value
Anterior upper	Side	0.0227	− 0.1881 to 0.2335	0.833	0.0227	− 0.1545 to 0.1999	0.802
	Age	0.0010	− 0.0042 to 0.0061	0.717	− 0.0011	− 0.0055 to 0.0033	0.629
	Sex	0.0077	− 0.2163 to 0.2318	0.946	0.0401	− 0.1510 to 0.2311	0.681
	BMI	0.1312	0.1052 to 0.1572	<0.001	0.1319	0.1058 to 0.1581	0.000
Posterior upper	Side	− 0.0794	− 0.6068 to 0.4478	0.768	− 0.0795	− 0.5529 to 0.3939	0.742
	Age	0.0036	− 0.0092 to 0.0165	0.583	0.0077	− 0.004 to 0.1951	0.200
	Sex	− 1.5914	− 2.1140 to − 1.0689	<0.001	− 1.6201	− 2.1307 to − 1.109	0.000
	BMI	0.1505	0.0755–0.2256	<0.001	0.1408	0.7096 to 0.2106	0.000
Total upper	Side	− 0.0568	− 0.6979 to 0.5842	0.862	− 0.0568	− 0.6191 to 0.5054	0.843
	Age	0.0045	− 0.0111 to 0.0202	0.568	0.0066	− 0.0073 to 0.0206	0.354
	Sex	− 1.5838	− 2.2347 to − 0.9329	<0.001	− 1.5802	− 2.1865 to − 0.9737	0.000
	BMI	0.2817	0.1947 to 0.3688	<0.001	0.2727	0.1898 to 0.3557	0.000
Anterior lower	Side	− 0.0332	− 0.2502 to 0.1838	0.764	− 0.0332	− 0.2220 to 0.1555	0.730
	Age	0.0083	0.0031 to 0.0135	0.002	0.0062	0.0014 to 0.0109	0.010
	Sex	0.1445	− 0.0854 to 0.3744	0.218	0.1214	− 0.0822 to 0.3249	0.243
	BMI	0.1153	0.0870 − 0.1435	<0.001	0.1126	0.0847 to 0.1404	0.000
Posterior lower	Side	0.1073	− 0.3560 to 0.5707	0.650	0.1073	− 0.3106 to 0.5252	0.615
	Age	0.0141	0.0029 to 0.0253	0.014	0.0155	0.005 to 0.0259	0.004
	Sex	− 0.8996	− 1.3788 to − 0.4204	<0.001	− 0.9793	− 1.4300 to − 0.5285	0.000
	BMI	0.1471	0.1098 to 0.2385	<0.001	0.1627	0.1010 to 0.2243	0.000
Total lower	Side	0.0742	− 0.5159 to 0.6644	0.805	0.0743	− 0.4411 to 0.5897	0.778
	Age	0.0223	0.0081 to 0.0365	0.002	0.0217	0.0088 to 0.0344	0.001
	Sex	− 0.7551	− 1.3751 to − 0.1351	0.017	− 0.8580	− 1.4138 to − 0.3021	0.002
	BMI	0.2895	0.2111 to 0.3679	<0.001	0.2753	4.8318 to 9.1715	0.000
Total orbital fat	Side	0.0174	− 0.9005 to 0.9354	0.970	0.0174	− 0.6922 to 0.7270	0.962
	Age	0.0269	0.0047 − 0.0491	0.017	0.0283	0.0105 to 0.0459	0.002
	Sex	− 2.3389	− 3.2679 to − 1.4099	<0.001	− 2.4381	− 3.2033 to − 1.6728	0.000
	BMI	0.5713	0.4578 to 0.6847	<0.001	0.5480	0.4433 to 0.6527	0.000
Orbital cavity volume	Side	0.0681	− 0.6625 to 0.7989	0.855	0.0682	− 0.5102 to 0.6465	0.817
	Age	0.0213	0.0037 to 0.0390	0.018	0.0304	0.0160 to 0.0448	0.000
	Sex	− 3.0511	− 3.7233 to − 2.3789	<0.001	− 3.2274	− 3.8511 to − 2.6036	0.000
	BMI	0.2440	0.1413 to 0.3467	<0.001	0.2168	0.1314 to 0.3021	0.000
Globe	Side	0.1201	− 0.1395 to 0.3796	0.365	0.1201	− 0.1180 to 0.3581	0.323
	Age	− 0.0108	− 0.0169 to − 0.0045	0.001	− 0.0086	− 0.0145 to − 0.0026	0.004
	Sex	− 0.7562	− 1.0152 to 0.4972	<0.001	− 0.6891	− 0.9458 to − 0.4324	0.000
	BMI	0.0292	− 0.0088 to 0.0672	0.132	0.0312	− 0.0039 to 0.6629	0.082
AP position	Side	− 0.008	− 0.0282 to 0.0122	0.439	− 0.0080	− 0.0261 to 0.0101	0.386
	Age	− 0.0009	− 0.0013 to − 0.0003	<0.001	− 0.0011	− 0.0015 to − 0.0006	0.000
	Sex	0.0047	− 0.0168 to 0.0263	0.665	0.0140	− 0.0055 to 0.3351	0.159
	BMI	0.0083	0.0056 to 0.0112	<0.001	0.0090	0.0063 to 0.0116	0.000
Vertical position	Side	− 0.0004	− 0.0071 to 0.0062	0.896	− 0.0004	− 0.0069 to 0.0061	0.895
	Age	0.0001	− 0.0001 to 0.0002	0.197	0.0001	− 0.00002 to 0.0003	0.094
	Sex	− 0.0051	− 0.0121 to 0.0019	0.156	− 0.0062	− 0.0132 to 0.0008	0.086
	BMI	− 0.0005	− 0.0015 to 0.0004	0.254	− 0.0007	− 0.0016 to 0.0003	0.181
